# Metastasis of Follicular Thyroid Carcinoma to Skull Base: A Case Report

**DOI:** 10.7759/cureus.28571

**Published:** 2022-08-30

**Authors:** Chim M Yang

**Affiliations:** 1 Otolaryngology-Head and Neck Surgery, Western Reserve Hospital, Cuyahoga Falls, USA

**Keywords:** lenvatinib, pembrolizumab, cancer immunotherapy, skull base tumors, follicular carcinoma of the thyroid

## Abstract

Follicular thyroid carcinoma is a well-differentiated thyroid neoplasm that is classified into widely invasive or minimally invasive carcinoma. Vascular invasion is often characteristic of follicular thyroid carcinoma. There is an estimated 6-20% incidence of distance metastasis with the bone being the most common followed by the lungs and then the lymph nodes. Metastatic spread to the skull base is rare. This case report will cover a patient who presented with follicular thyroid carcinoma and cranial neuropathy and was found to have metastasis to the skull base.

## Introduction

Thyroid cancer accounts for 3.8% of new cancer cases and is the ninth most common cancer in the United States [1]. Thyroid cancer is divided into undifferentiated and differentiated cancer. The undifferentiated cancers include anaplastic thyroid carcinoma and medullary thyroid carcinoma, while the differentiated cancers include follicular thyroid carcinoma and papillary thyroid carcinoma. Within the subset of differentiated thyroid cancer, follicular thyroid carcinoma accounts for up to 10-15% [2]. Follicular thyroid carcinoma is a malignant neoplasm of the follicular cells made up of cuboidal cells. The cells have invasive properties specifically towards vascular structures. The most common mutation is a RAS point mutation, while one-third of patients may exhibit a peroxisome proliferator-activated receptor gamma rearrangement [3]. There is an estimated 6-20% incidence of distant metastasis with follicular thyroid carcinoma [4]. Metastasis to the bone occurred at 42%, followed by the lungs at 33%, and then to the lymph nodes at 8% [5]. Follicular thyroid carcinoma metastasis to the skull base is rare but warrants a thorough workup when a patient presents with lower cranial neuropathy.

## Case presentation

**Figure 1:**
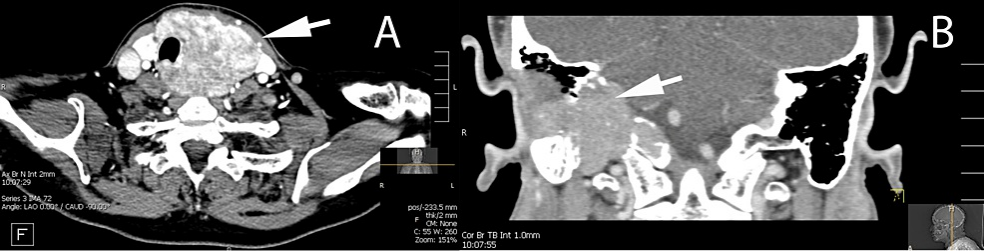
Computed tomography with contrast of neck and right skull base. (A) Avidly enhancing mass of the left thyroid gland (arrow) with tracheal deviation to the right. (B) Avidly enhancing mass centered in the right jugular foramen (arrow) with regional osseous destruction of the petrous and mastoid temporal bone.

A 57-year-old female presented to the otolaryngology practice with progressive dysphagia, hoarse voice, weakness of her right shoulder, and a goiter. In-office nasopharyngoscopy examination demonstrated a paralyzed right true vocal cord. Blood work obtained shows thyroid stimulating hormone of 0.2 mcU/mL (normal range: 0.5-5.0 mcU/L), free T4 of 1.10 ng/dL (normal range: 0.7-1.90 ng/dL), and carcinoembryonic antigen of 2.7 ng/dL (normal range: <3 ng/dL). Given the dysphagia, paralyzed right true vocal cord, and goiter, a computed tomography scan of her neck with contrast and modified barium swallow was obtained. Computed tomography scan of her neck with contrast revealed a large left thyroid lobe mass (Figure 1A) as well as enhancing soft tissue mass at the right jugular foramen (Figure 1B).

**Figure 2:**
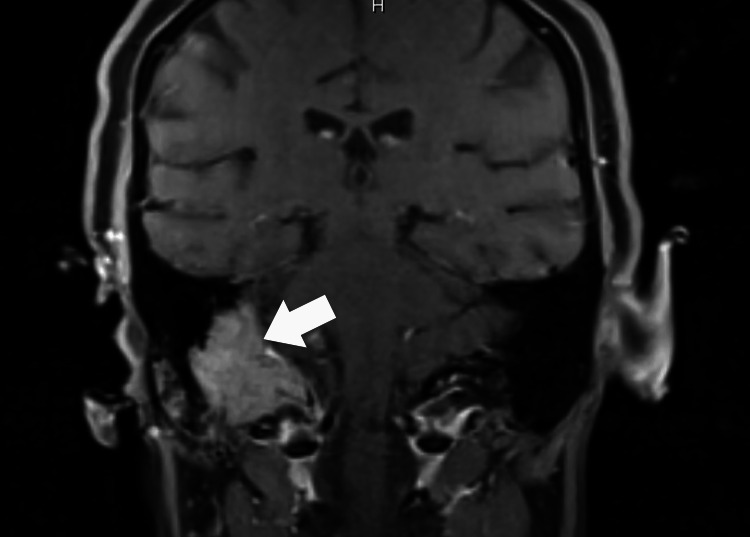
Coronal magnetic resonance imaging with gadolinium contrast of the skull base The arrow points to right skull base with enhancing lesion.

The modified barium swallow revealed moderate pharyngeal dysphagia. The efficiency of her pharyngeal clearance showed asymmetric bolus flow and narrowing through her proximal esophagus in the anterior-posterior view consistent with the left thyroid mass with tracheal deviation. She was placed on a full liquid diet to prevent aspiration. A magnetic resonance imaging of the brain with gadolinium was obtained to better delineate the enhancing soft tissue mass at the right jugular foramen. The magnetic resonance Imaging of the brain with gadolinium showed a locally destructive avidly enhancing mass centered at the right jugular foramen (Figure 2).

**Figure 3:**
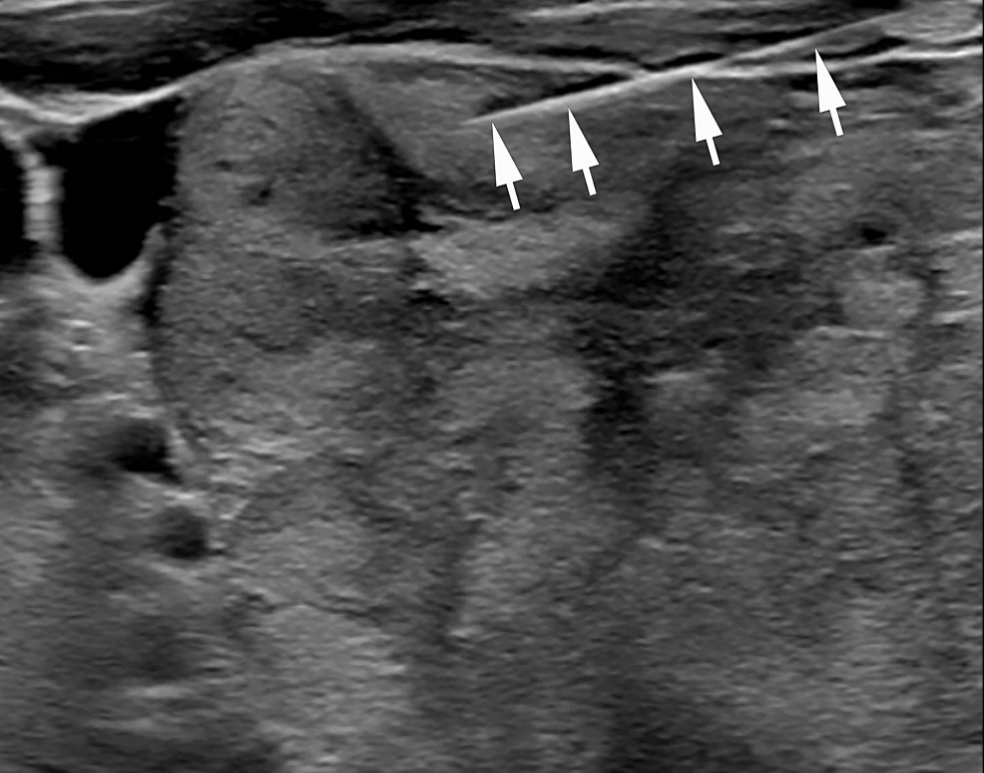
Transverse view of the ultrasound-guided fine needle aspiration of the left thyroid lobe. The arrows are pointing to the fine needle.

Fine needle aspiration was performed under ultrasound guidance to sample the left thyroid tissue and the results were consistent with follicular neoplasm (Figure 3).

**Figure 4:**
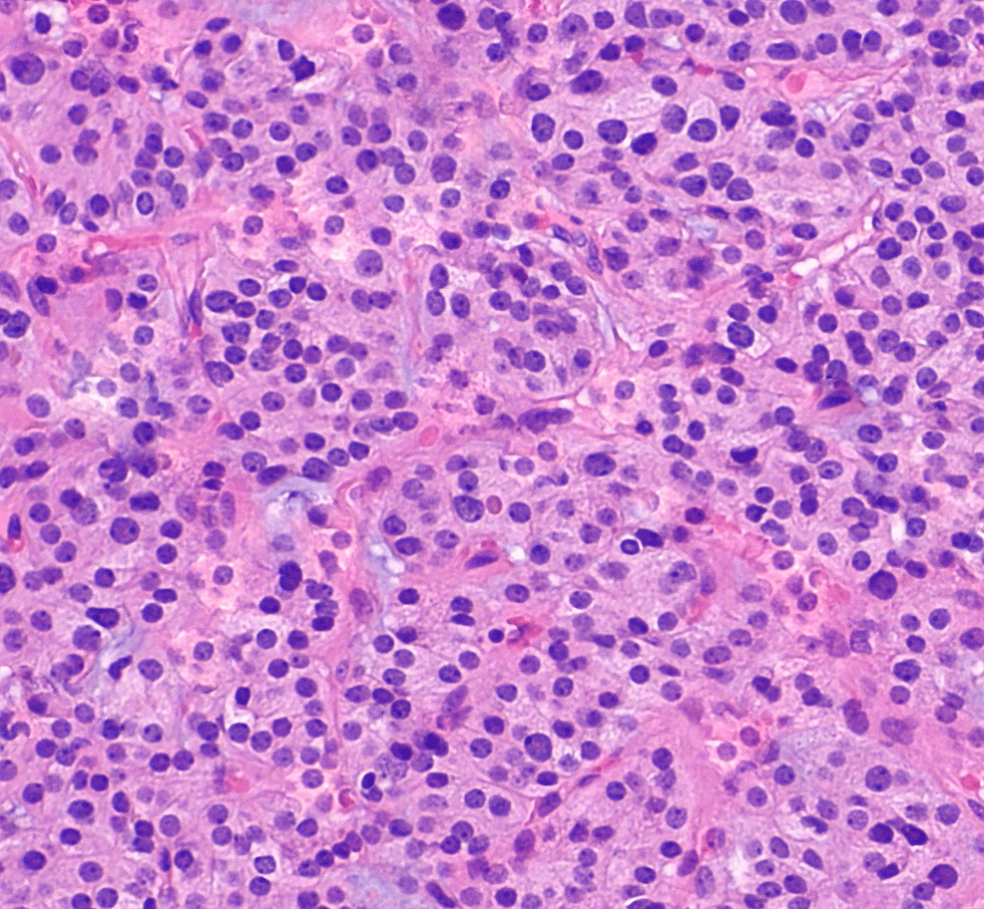
Follicular thyroid carcinoma histology Follicles in a trabecular pattern are visible under 400x high power magnification. There are no nuclear features of papillary thyroid carcinoma, squamous metaplasia, or psammoma bodies.

The patient was taken into the operating room for a total thyroidectomy and mastoidectomy for a biopsy of the soft tissue mass at the right jugular foramen. Under general anesthesia, a cortical mastoidectomy was performed. There were obvious tumor cells present in the air cells below the labyrinth. Biopsies were taken and sent for frozen section analysis which revealed thyroid tissue. The periosteum was closed along with the dermis. Attention was then turned to performing the total thyroidectomy. Once the dissection proceeded down to the thyroid, it was clear that the tumor extended along the right superior thyroid vein to right the jugular vein. The decision was made to abort the planned total thyroidectomy due to the high risk of recurrent laryngeal nerve injury. An incisional biopsy was instead obtained from the thyroid mass. The recurrent laryngeal nerves were stimulated at the carotid sheaths on both sides at 0.8 mA and confirmed to be intact. The surgical site was closed, and the patient was kept overnight for observation and discharged on postoperative day one. The biopsy results show both specimens are morphologically similar and consist of bland thyroid tissue with follicular morphology (Figure 4).

Given the clinically invasive features, the findings were consistent with widely invasive follicular carcinoma. The patient's case was discussed at the multidisciplinary tumor board due to the unresectable nature of the disease. The recommendation was made to start palliative intensity-modulated radiation therapy to the right skull base with 3700 Gy in 10 daily fractions and then lenvatinib plus pembrolizumab immunotherapy in combination afterward.

## Discussion

Follicular thyroid carcinoma is classified into widely invasive or minimally invasive carcinoma. The overall 10-year survival for minimally invasive follicular thyroid carcinoma is reported to be 98%, while invasive follicular thyroid carcinoma is reported to be 80% [6]. Extrathyroidal invasion, age greater than 45 years, distant metastasis, and tumor size are poor prognostic risk factors [7]. Management of metastatic follicular thyroid carcinoma is done with surgery and radioiodine ablation. Success is largely dependent on surgical resection and whether the tumor tissue takes up the radioiodine.

Given the disease burden on the patient, surgical resection and radioiodine ablation were limited options. Follicular thyroid carcinoma metastasizes through angioinvasion with the lung being the most common location but rarely to the skull base [4]. When the jugular foramen is affected, patients may present with loss of sensation in their posterior ipsilateral tongue, hoarseness of their voice, dysphagia, and drooping of the ipsilateral shoulder given the cranial nerve involvement of IX, X, and XI.

Lenvatinib is a multiple kinase inhibitor that is approved for the treatment of differentiated thyroid cancer unresponsive to radioactive iodine or unresectable advanced disease [8]. In a multicenter randomized placebo-controlled phase three trial of lenvatinib with 392 participants with radioactive resistant differentiated thyroid cancer, the study showed a median progression-free survival of 18.3 months with a response rate of 64.8% when compared to the placebo arm [9]. Pembrolizumab is a monoclonal immunoglobulin G antibody that blocks the interaction between programmed cell death protein 1 and its ligand on the surface of T-cells [10]. Pembrolizumab’s antitumor activity is effective in head and neck cancer. A study done by Dierks et al. showed a combination of lenvatinib and pembrolizumab in the treatment of metastatic advanced thyroid cancer had a response of 66% complete remission and median overall survival of 18.5 months [11].

In the review of the current literature, 29 cases of skull bases metastasis from follicular thyroid carcinoma have been reported including this case [12-18]. There is a female predominance as in our case report [13]. On radiological imaging, local invasion of surrounding tissue and bony destruction were common findings. Often times the radiological imaging may be mistaken for chondrosarcoma, chordoma, or melanoma [16]. The most common reported symptom of skull base metastasis were cranial nerve dysfunctions such as dysphagia, shoulder weakness, or tongue weakness. Skull base metastasis from follicular thyroid carcinoma is rare but should be considered in patients with cranial nerve dysfunction, supported by radiological findings, and clinical assessment of thyroid neoplasm.

## Conclusions

Here we present a case of widely invasive follicular carcinoma with metastasis to the skull base. Patients who present with follicular thyroid carcinoma and have cranial neuropathy warrant close attention and proper workup. Management of metastatic follicular thyroid carcinoma is done with surgery and radioiodine ablation. Success is largely dependent on surgical resection and whether the tumor tissue takes radioiodine avid. When the disease burden prevents these options from becoming successful, targeted radiation therapy and lenvatinib with pembrolizumab are therapeutic options.
